# Use of the Sesqui-Chloride of Chromium in Sensitive Dentine

**Published:** 1871-01

**Authors:** Geo. S. Yingling


### ARTICLE IV.

### *Use of the Sesqui-Chloride of Chromium in Sensitive Dentine.*

By Geo. S. Yingling, M. D.

While on a visit to my home in Tiffin, Ohio, I was requested by Dr. Bricker, of that city, to procure for him on my return to Baltimore, a small quantity of the Sesqui-Chloride of Chromium, for the purpose of applying it to a cancerous breast.

He stated that its action, when applied for the removal of cancerous growths, was peculiar; accomplishing the result thoroughly by a sort of disintegrating or crumbling away process and entirely painless.

With some difficulty I procured the chromium, but before sending it to Dr. Bricker, I showed it to my preceptor, Jas. H. Ludwig, M. D., D. D. S., and made known to him the name of the article, and the purpose for which it was procured.

Having at that time an engagement with a patient, whose teeth were exceedingly sensitive, and being somewhat at a loss what agent to use in order to relieve the hypersensitiveness in the particular tooth which he intended to fill then, most of the agents having already been used unfavorably, the idea occured to him that the chromium might effect the purpose desired.

The following is an account of Dr. Ludwig's case in his own language, which I apprehend will be both useful and interesting to the profession:

"The tooth I selected for testing the merits of the sesqui-chloride of chromium, as an agent for the treatment of inflamed dentine, was but slightly decayed, the caries having penetrated but little further than the enamel.

"The sensitiveness was of such an exalted character that cold fluids and even the cool air would affect it painfully.

The application of an instrument would produce extreme torture, and I found it simply impossible to excavate it without inflicting upon the patient intense suffering, unless I could first destroy the morbid sensitiveness by the application of some medicinal agent.

"For this purpose I tried in succession, carbolic acid, chloride of zinc, chloroform and chloral hydrate, but without favorable results. In each case the application of the above agents was followed by great pain.

"When, therefore, Dr. Yingling mentioned to me the action of the sesqui-chloride of chromium on cancerous growths, viz: that it caused the diseased part to come away by a kind of crumbling process, and without pain, the thought occured to me that it might act favorably upon the case I then had in hand, if not in all cases where the use of such an agent became necessary.

"I accordingly applied a small quantity of the sesqui-chloride in a semi-fluid condition to the cavity of decay. Its application was followed by some pain, which was however of short duration, and may have been owing to the previous applicatiou of other agents, or to the too concentrated form of the chromium. The chromium being of a green color, the dentine was stained and presented the same hue.

"The surplus chromium was now removed, the cavity excavated and properly shaped *without any pain*, but there being some discolorization left, I concluded to defer the filling of the cavity with gold for several weeks, in order to see what effect time would have upon the texture and color of the cavity, keeping it in the meantime filled with Hill's stopping.

"At the expiration of about three weeks, I removed the temporary filling and found the discoloration had disappeared, and the dentine perfectly firm, and free from sensitiveness.

"I have used it in other cases, but in these cases the sensitiveness was not great, and other agents probably would have done as well, but in all instances where I employed it favorable results followed.

"I also employed it in a case of *morbid growth of the gums.* The gums were extremely vascular, discharged a very offensive matter, and had in them a constant burning sensation. The patient, formerly a healthy woman, had, by living on the plains, where she was in a measure deprived of fresh meat and vegetables, contracted scorbutus, and this morbid growth was no doubt owing to the existing cachexia.

"I applied the chromium a number of times, but it did not realize my expectations, while Nitrate of Silver acted kindly and efficiently."

Being a student at the Baltimore College of Dental Surgery at the time, and being called upon to operate in the Infirmary, upon teeth more or less sensitive, I took a small quantity of the chromium with me, and made known to Dr. Clark, our gentlemanly demonstrator of operative dentistry, its nature and action upon diseased growths. He kindly suggested to me the name of Mr. King, one of the students, as a suitable person upon whom to experiment, he having teeth very much decayed and in an extremely sensitive condition.

Mr. King very willingly consented to have an application of the chromium made to his teeth, the result being most satisfactory, as will be seen by a history of the case as given by himself, and which I append.

"My teeth decayed early in life, and I was in consequence obliged to endure many weary and tedious operations in order to rescue them from the ravages of the destructive agent at work in them. A history of my case, may, therefore, be the means of lessening, if not entirely obviating in others, the torments which I have endured for the last ten years on account of the hypersensitiveness of my teeth. When a youth my teeth were comparatively good and I had each one attended to as deemed necessary by my dentist. But owing to neglect the decay in them progressed rapidly. Beginning at the central incisors it attacked the greater part of the dentine, leaving the remaining portion of this tissue in such an extremely sensitive condition that thirst was often more preferable than a drought of cold water, cold air, or any cold substance would affect them acutely; and I have often refrained from a hearty laugh for fear of the air coming in contact with them, and giving me the most exquisite pain.

"From the centrals, its ravages continued around the entire arch, until all the superior teeth were invaded. They were no longer of any benefit or comfort to me–but a burthen, and I came to the conclusion that mine was an unusual case, so aggravated that there was no relief for me but to have the offending members extracted. About this time I commenced the study of dentistry, and began to investigate the case for myself. I tried in succession the various agents commonly used in such cases, but to my utter discomfort, found every one to end in failure.

"While attending the Baltimore College of Dental Surgery this winter, I had them examined by several of the Faculty who, in view of their decayed and hypersensitive condition, advised their extraction.

"Being thus convinced of their irreparable condition, and therefore taking but little interest in their future preservation, I permitted them to be experimented upon by Dr. Yingling, a student in the College, who was desirous of testing a new agent—the sesqui-chloride of chromium—upon them.

"The Doctor accordingly made an application on one of the incisors, and to my great astonishment and relief it acted like a charm. I could afterward apply any cold substance to them without any painful sensation whatever, the same being true also in regard to the excavator.

"Had I met the Doctor and his chromium a few years ago, I might now have a good set of my *own* teeth, borrowing the advertisement of operative dentistry, which they would have displayed on account of the decay being mostly on the labial surfaces.

"I hope soon to see this agent extensively used by all operators who would perform comparatively painless operations, for I feel that from its favorable results in my case, that it will prove an efficient addition to the list of agents already in use for the treatment of sensitive dentine."

I applied the chromium to an incisor in Mr. King's case, by mixing it with water to the consistency of cream, carrying it to the cavity of the tooth upon a pledget of cotton. The tooth which before the application was so sensitive, afterwards was found to bear the burr-drill without pain; the sensitiveness did not return for three weeks, and then but slightly.

Prof. Gorgas, and many of the students have since used the chromium with equally good results.

The chromium when applied to dentine, colors it dark green, but does not penetrate far into its substance and may be easily removed with the excavator, or, if let alone, will disappear in a few days. The enamel is not affected by the stain.

I should be pleased to have the members of the profession try this agent on sensitive dentine and report their success to the journals.

